# The Application of the Lymphoma International Prognostic Index to Predict Venous Thromboembolic Events in Diffuse Large B-Cell Lymphoma Patients

**DOI:** 10.3389/fonc.2021.677776

**Published:** 2021-05-28

**Authors:** Hikmat Abdel-Razeq, Mohammad Ma’koseh, Rashid Abdel-Razeq, Rula Amarin, Alaa Abufara, Razan Mansour, Mohammad Manasrah, Mohammad Al-Rwashdeh, Rayan Bater

**Affiliations:** ^1^ Department of Internal Medicine, King Hussein Cancer Center, Amman, Jordan; ^2^ School of Medicine, University of Jordan, Amman, Jordan; ^3^ Department of Medicine, Istishari Hospital, Amman, Jordan

**Keywords:** International Prognostic Index (IPI), thrombosis, NHL (non-Hodgkin lymphoma), lymphoma, DLBCL

## Abstract

**Background:**

Venous thromboembolic events (VTE) are commonly encountered in patients with lymphoma. Several risk assessments models (RAM) had attempted to identify higher risk patients with varying success. The International Prognostic Index (IPI) is a clinicopathological tool developed to help predict both response to treatment and prognosis of patients with diffuse large B-cell lymphoma (DLBCL).

**Objective:**

In this study, we utilize the IPI index to identify group of patients with DLBCL at higher risk for VTE.

**Patients/Methods:**

Patients with pathologically-confirmed diagnosis of DLBCL and with image-confirmed VTE, treated and followed at our institution were included. Rates of VTE was calculated for each risk category.

**Results:**

A total of 373 patients, median age 49 (range: 18-90) years were included. VTE were reported in 56 (15.0%) patients; 51 (91.1%) had active disease while 29 (51.8%) were ambulatory at time of VTE diagnosis. VTE rates were particularly high among patients with poor performance status (26.2%, P=0.028) and high LDH (19.0%, P=0.023). Applying the age-adjusted IPI separated patients into two risk categories; VTE were diagnosed in 9.7% in patients with “low and low-intermediate” scores compared to 19.8% in patients with “high and high-intermediate” scores, P=0.020.

**Conclusions:**

The original IPI and its modified versions, routinely used at diagnosis as a prognostic and predictive tool for patients with DLBCL, can also be utilized to define high risk patients for VTE; the risk of whom might be high enough to recommend thromboprophylaxis even in the ambulatory settings. More work is needed to refine and improve currently available RAMs.

## Introduction

Venous thromboembolic events (VTE) are commonly encountered in patients with cancer (1). Compared to most patients with other tumors, it is widely believed that patients with non-Hodgkin’s lymphoma (NHL) are at even higher risk for VTE, especially those undergoing chemotherapy where VTE rate may reach 15% or higher (2, 3). Such episodes can be fatal, may delay chemotherapy and important invasive interventions and may lead to chronic complications that may negatively affective the quality of life (QoL) of survivors (4).

Diffuse large B-cell Non-Hodgkin’s lymphoma (DLBCL) is one of the aggressive and the most common histologic subtype of non-Hodgkin lymphoma (NHL), accounting for almost 25% of NHL encountered among adults worldwide (5, 6). Its incidence increases with age; the median age at presentation is 64 years but appears to be younger for other ethnicities, like African-American (7).

Thromboprophylaxis for surgical and medical patients, including those with cancer, admitted to the hospital with acute medical problem or for surgical intervention is widely practiced and improving (8). However, patients with cancer, including those receiving infusion chemotherapy, are treated in the outpatient settings. Many studies had shown that majority of cancer patients develop their VTE while in ambulatory settings where VTE prophylaxis is not routinely practiced (9).

Several risk assessments models (RAM) for chemotherapy-associated thrombosis attempted to identify patients at higher risk for VTE (10–13). Due to poor discriminatory performance, particularly when studied on a single disease cohort, most of such models have proven to be of limited clinical utility (14–17). Given these limitations, Antic et al. developed and validated a new model, known as the Thrombosis Lymphoma (ThroLy) score, to predict thromboembolic events in lymphoma patients (18, 19).

The International Prognostic Index (IPI) is a simple tool utilizes clinicopathological parameters [age, lactate dehydrogenase (LDH), number/sites of involvement, stage and patients’ performance status (PS)], developed to aid predict both response to treatment and prognosis of patients with newly diagnosed DLBCL. The age-adjusted IPI is a modified version with both age and extra nodal disease were dropped, while the Revised IPI (R-IPI) utilized the same 5 factors but grouped the patients into 3 (not 4) risk levels (very good, good and poor) (20, 21).

In this study, we utilize the IPI index, and its modified versions, to identify group(s) of patients with DLBCL at higher risk for VTE to possibly recommend VTE prophylaxis even in ambulatory settings.

## Patients and Methods

Consecutive patients with pathologically-confirmed diagnosis of DLBCL, treated and followed at our institution were enrolled. Hospital databases and medical records were searched for patients with image-confirmed VTE from the date of DLBCL diagnosis through the initial treatment but didn’t include relapsed patients. Deep vein thrombosis (DVT) was diagnosed by doppler ultrasound and was used for symptomatic patients; routine screening for VTE was not done. Pulmonary embolism (PE) was diagnosed by computed tomography (CT) angiography. We searched patients’ medical records for all clinical and laboratory data related to VTE including patients’ age, disease stage, serum lactate dehydrogenase (LDH), Eastern Cooperative Oncology Group (ECOG) performance status (PS) and number and sites of extra nodal disease. We also identified patients with bulky disease (tumor mass >10 cm) and those with mediastinal involvement. Methods, duration and complication of anticoagulation, when used for patients with VTE, were also recorded.

Given the retrospective nature of the study and the lack of personal or clinical details of participants that compromise anonymity, consent was waived and the study was approved by King Hussein Cancer Center Institutional Review Board (IRB).

### Statistical Analysis

Descriptive statistics including the frequency, mean, and standard deviation (SD) are presented for normally distributed variables. Medians and the standard errors (SE) with interquartile ranges (IQR) were used. We compared the rate of VTE according to the presence or absences of each of the 5 factors including in the IPI. VTE rates were also compared according to risk levels (low, low-intermediate, high-intermediate and high) in the original and revised IPI or (very good, good and poor risks) in the age-adjusted IPI. We then combine each IPI scores into two risk levels and compared VTE rates accordingly. A p-value < 0.05 was regarded as statistically significant. Version 9.4 of SAS software (SAS Institute Inc., Cary, NC) was utilized.

## Results

Between 2006 and 2018, a total of 373 patients (52.5% males) were identified. Median age was 49 (range: 18-90) years, with almost a third (n=113, 30.3%) were above 60 years. All had diffuse large B-cell lymphoma; 196 (52.5%) with stage III-IV, 189 (50.7%) with elevated LDH, 90 (24.1%) with ≥ 1 site of extra nodal involvement and 113 (30.3%) with bulky disease. At time of VTE diagnosis, most of the patients were fully ambulatory with only 61 (16.4%) had an ECOG performance status of 2-4. Except for 17 (4.6%) patients who were treated with less aggressive regimens, all patients were treated with a unified chemotherapy regimen consists of rituximab, cyclophosphamide, doxorubicin, vincristine and prednisone (R-CHOP); 273 (73.2%) achieved complete remission (CR) while 66 (17.7%) had additional (salvage) chemotherapy, some with autologous stem cell transplantation, **Table 1**.

**Table 1:** Patients’ characteristics (n = 373).

**Characteristics**		**Number**	**Percentage**
**Age (Years)**	Median: 49		
	Range: 18-90		
**Sex**	Male	196	52.5
	Female	177	47.5
**Smoking**	Active smoker	101	27.1
	Prior smoker	43	11.5
	Never smoked	221	59.2
	Unknown	8	2.1
**ECOG Performance Status (PS)**	0-1	312	83.6
	2	29	7.8
	3-4	32	8.6
**Stage at diagnosis**	I	87	23.3
	II	90	24.1
	III	38	10.2
	IV	158	42.4
**Bulky Disease**		113	30.3
**Initial Chemotherapy**	R-CHOP	356	95.4
	CHOP	8	2.1
	R-CVP	5	1.3
	R-CHOP+HDMTX	2	0.54
	DA-EPOCH-R	2	0.54
**Response to Treatment**	Complete Response (CR)	273	73.2
	Partial Response (PR)	35	9.4
	Disease Progression (DP)	50	13.4
	NA*	15	4.0
**Salvage chemotherapy**		66	17.7

*NA, Data not available; Dead: 12, Lost follow-up: 3.

ECOG, Eastern Cooperative Oncology Group; R-CHOP, Rituximab, Cyclophosphamide, Doxorubicin, Vincristine and Prednisone; CHOP, Cyclophosphamide, Doxorubicin, Vincristine and Prednisone; R-CVP, Rituximab, Cyclophosphamide, Vincristine and Prednisone; HDMTX, High-dose Methotrexate; DA-EPOCH-R, Dose adjusted, Etoposide, Prednisone, Vincristine, Cyclophosphamide, Doxorubicin and Rituximab.

Venous thromboembolic events were objectively confirmed in 56 (15.0%) patients. Deep vein thrombosis involved the upper and lower extremities in 15 (26.8%) each, 5 (8.9%) had both DVT and PE while another 15 (26.8%) had isolated PE without apparent DVT. Majority (n=51, 91.1%) of the patients had their clot diagnosed while on active treatment with chemotherapy and 29 (51.8%) were ambulatory with no recent hospitalization, **Table 2**. All patients were treated with low molecular weight heparin (LMWH) that was complicated by 3 episodes of bleeding; 2 were major. Two other patients experienced recurrence of their clot and one patient had thrombocytopenia.

**Table 2:** Venous thromboembolism (n=56).

Clinical variables	Number of patients	Percentage
**VTE Site**		
Lower extremity alone	15	26.8
Upper extremity alone	15	26.8
PE	15	26.8
DVT and PE	5	8.9
Others	6	10.7
**Disease status at time of VTE**		
Active disease	51	91.1
Disease-Free	5	8.9
**Relation to Chemotherapy**		
Before Chemotherapy	19	34.0
Within 30 days from chemotherapy	37	66.1
More than 30 days	0	0
**Relation to hospitalization**		
Not related (ambulatory)	29	51.8
Within 30 days of hospitalization	27	48.2

DVT, Deep Vein Thrombosis; PE, Pulmonary Embolism; VTE, Venous Thromboembolism.

Venous thromboembolic events rate was particularly high (26.2%) among patients with poor performance status (ECOG PS: 2-4) compared to a rate of 12.9% among patients with good performance status (ECOG PS: 0-1), P=0.028. Additionally, compared to patients with normal LDH, patients with elevated levels had higher rate of VTE; 19.0% compared to 9.4%, p=0.023. Age (≤60 versus >60years), disease stage (I & II versus III & IV) and the presence of extranodal disease had no significant impact on VTE rates. VTE rates in relation to clinical components of the IPI are detailed in **Table 3**.

**Table 3:** VTE rates according to components of the International Prognostic Index (IPI).

**Criteria**		**Patients**		**Patients with VTE**		**P-value**
		**Number**	**Percentage**	**Number**	**Percentage**
ECOG Performance Status (PS)*	0-1	312	83.6	40	12.9	0.028
	2-4	61	16.3	16	26.2	
**LDH^**	Normal	180	48.3	17	9.4	0.023
	Elevated	189	50.7	36	19.0	
**Age** (years)	≤60	260	69.7	37	14.2	0.583
	>60	113	30.3	19	16.8	
**Stage**	I-II	177	47.5	24	13.6	0.520
	III-IV	196	52.5	32	16.8	
**Extranodal disease**	0,1 site	283	75.9	41	14.5	0.666
	>1 site	90	24.1	15	16.6	

VTE, Venous Thromboembolic Events; ECOG, Eastern Cooperative Oncology Group; LDH, Lactate Dehydrogenase.

*Data not available on 2 patients, ^Data not available in 4 patients.

**Figure 1:**
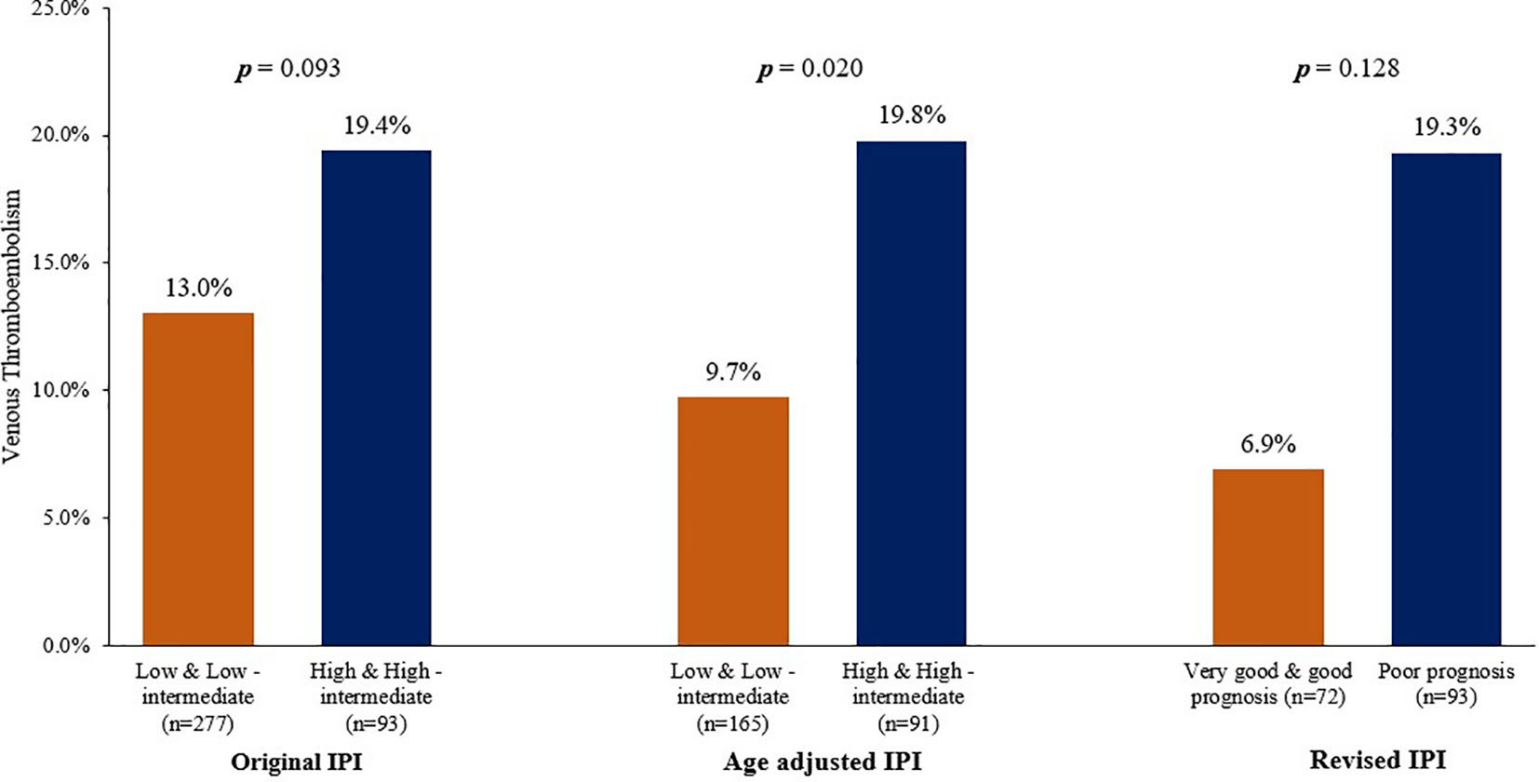
Venous Thromboembolic Event rates according to different IPI versions.

We evaluated VTE rates according to the original IPI and its modified versions. In the original IPI, highest VTE rate (25.0%) was among a group of 40 patients with “high” IPI scores (score: 4-5), compared to rates of 10.8%, 16.4% and 15.1% in patients with “low”, “low-intermediate” and “high-intermediate” IPI scores, respectively (**Table 4**). In total, 36 (13.0%) of 277 patients with “low and low-intermediate” IPI scores (score: 0-2) had VTE, compared to 18 (19.4%) of 93 patients with “high and high-intermediate” IPI scores (score: 3-5), p=0.093, **Figure 1**.

**Table 4:** VTE rates according to IPI versions.

**Risk Factors and Index**	**Parameter**	**Patients**		**Patients with VTE**		**P-value**
		**Number**	**Percentage**	**Number**	**Percentage**
Original IPI*	0-1= Low	167	44.8	18	10.8	0.252
	2 = Low-intermediate	110	29.5	18	16.4	
	3= High-intermediate	53	14.2	8	15.1	
	4-5= High	40	10.7	10	25.0	
**Age-adjusted IPI** ^^,#^	0= Low	73	28.1	5	6.8	0.153
	1= Low-intermediate	92	35.4	11	12	
	2= High-intermediate	81	31.2	15	18.5	
	3= High	10	3.8	3	30.0	
**Revised-IPI**^	0= Very good	72	19.3	5	6.9	0.139
	1,2= Good	204	54.7	30	14.7	
	3-5= Poor	93	24.9	18	19.3	

VTE, Venous Thromboembolic Events; IPI, International Prognostic Index.

*Data not available on 3 patients; ^Data not available on 4 patients; ^#^Out of 260 patients aged ≤ 60 years.

Using the age-adjusted IPI, VTE were diagnosed in 5 (6.8%), 11 (12.0%), 15 (18.5%) and 3 (30.0) % among patients with “low”, “low-intermediate”, “high-intermediate” and “high” age-adjusted IPI, respectively (**Table 4**). Similarly, when patients were grouped into two risk categories; VTE rates were 9.7% in patients with “low and low-intermediate” age-adjusted IPI compared to 19.8% in patients with “high and high-intermediate” age-adjusted IPI, p=0.020, **Figure 1**.

The revised-IPI score was also tested; VTE were diagnosed in 5 (6.9%), 30 (14.7%) and 18 (19.3%) among patients with very good (score=0), good (score=1, 2), and poor prognosis group (score=3-5), respectively (p=0.022), **Table 4**. However, VTE rate was not statistically different between the “poor prognosis” group (n=93, VTE rate: 19.3%) and the combined “good” and “very good” prognosis group (n=276, VTE rate: 12.7%), p= 0.128.

### Discussion

To our knowledge, this is the first attempt to utilize the IPI as a tool to assess the risk of VTE among patients with NHL. The clinical and pathologic features included in the IPI are by default lymphoma-specific and its association with higher risk of VTE is both understandable and predictable. Several previous studies addressed thromboprophylaxis in ambulatory cancer patients utilizing a risk stratification approach, and mostly were in patients with mixed tumors that occasionally included variable subtypes of lymphoma (10, 12). However, none took into consideration the many special features of lymphomas that can affect VTE rates. The addition of some biomarkers like D-Dimer or the cell adhesion molecule P-selectin (sP-selectin) in a modified version of Khorana RAM did not help, either (22).

More recently, Antic and colleagues introduced a new risk assessment model specifically for patients with different types of lymphomas. The model included history on prior venous or arterial thrombosis (including myocardial infarction and stroke), obesity, poor PS, extranodal disease, mediastinal involvement, low neutrophil counts and low hemoglobin. Based on the risk model scores, patients were divided to three risk groups: low (score 0-1), intermediate (score 2-3), and high (score >3). For patients at risk (intermediate and high-risk scores), the model has a negative predictive value of 98.5%, positive predictive value of 25.1%, sensitivity of 75.4%, and specificity of 87.5%. However, its adoption in clinical practice has been limited and several other studies questioned its validity (23).

Our suggested model for risk assessment depends on data already collected, and using an index that is widely used to guide oncologists predict the outcome of lymphoma patients prior to their treatment. Additionally, it includes just one type of lymphoma; diffuse large B-cell, which is one of the most common aggressive subtypes.

Though sounds confusing, the presence of many versions of the IPI shouldn’t be a problem for assessing patients’ risk for VTE. In all studied versions of the IPI, a similar proportion (almost 25%) of the total number of patients included, have almost similar high rates of VTE (19-20%).

Another version of the IPI, introduced by the National Comprehensive Cancer Network (NCCN-IPI), developed to further refine the importance of age and LDH as continuous variables; both were given different weights in the index based on their actual level. However, calculation is cumbersome and the index failed to gain popularity among practicing oncologists (24, 25).

Both, the IPI and the R-IPI scores were not significantly associated with VTE occurrence. The age-adjusted IPI score was the only score associated with VTE, probably a function of the ECOG performance status and LDH. Giving the availability of its components and the simplicity of its calculation, its utilization in clinical practice worth further consideration.

Our study highlights several issues related to the risk of VTE in patients with DLBCL. First, VTE risk is highest while the patient having an active disease. Second, even ambulatory patient with no recent hospitalization can be at risk for VTE. Third, almost a quarter of the patients are classified as having high or high-intermediate IPI score and the rate of VTE in this group is around 20%.

Our study can be an eye opener, first to validate our findings in a bigger study and then to include the high-risk patients identified by the IPI as a focus group for a randomized study to offer VTE prophylaxis for such patients while undergoing chemotherapy even in the ambulatory setting against a placebo-controlled arm. Given how common DLBCL is, a multi-institutional study can include thousands of patients to avoid selection bias and statistical problems. Though we, and many others, had identified other clinical features that might contribute to the risk of VTE like mediastinal mass, bulky disease, low hemoglobin and previous history of venous or arterial thrombosis, we believe simplicity is the goal here. Identifying a high-risk group for VTE based on existing index utilized in clinical practice for the same patients is a major advantage.

## Data Availability Statement 

The raw data supporting the conclusions of this article will be made available by the authors, without undue reservation.

## Ethics Statement

The studies involving human participants were reviewed and approved by King Hussein Cancer Center Institutional Review Board (IRB). Written informed consent for participation was not required for this study in accordance with the national legislation and the institutional requirements.

## Author Contributions

Conception and design: HA-R, MM’k. Provision of study materials or patients: MM’k, RA-R, RA. Collection and assembly of data: RA, AA, RM, MM, MA-R. Data analysis and interpretation: HA-R, MM’k, RB. All authors contributed to the article and approved the submitted version. Accountable for all aspects of the work: All authors.

## Conflict of Interest

The authors declare that the research was conducted in the absence of any commercial or financial relationships that could be construed as a potential conflict of interest.
